# Advanced Oxidation Processes of Organic Contaminants

**DOI:** 10.3390/toxics12080579

**Published:** 2024-08-09

**Authors:** Mingzhu Liu, Shengnan Zhang, Zunyao Wang

**Affiliations:** State Key Laboratory of Pollution Control and Resources Reuse, School of the Environment, Nanjing University, Nanjing 210023, China; 602022250029@smail.nju.edu.cn (M.L.); dg21250058@smail.nju.edu.cn (S.Z.)

Concern is growing about the occurrence of emerging organic contaminants in various eco-environments. Most pollutants are processed in our daily life by industrial production and released into the natural environment through different channels with their consumption [1]. Furthermore, this causes environmental pollution and exposure [2,3]. Several studies have reported the occurrence of these contaminants in drinking water [4]. Their toxic effects on organisms and the ecological environment should arouse vigilance [5,6]. Many compounds have endocrine disruptors that are often considered the biggest potential problem [7]. Additionally, the presence of multiple antibiotics will promote the production of antibiotic resistance genes and antibiotic-resistant bacteria [8]. Therefore, a variety of advanced oxidation processes (AOPs) should be used to degrade organic pollutants.

Biodegradation and physical adsorption can also be used to remove organic pollutants; however, these methods are not widely used due to poor removal efficiency and high operating costs [9,10]. AOPs stand out for their efficacy in treating a wide range of refractory organic contaminants and are suitable for the removal of multiple organics. The Fenton oxidation reaction, in which materials such as iron-based catalysts activate hydrogen peroxide to produce active substances that remove contaminants, has limitations in acidic systems [11]. Electrochemical oxidation utilizes an electric field to change the functional group of organic compounds, but it requires much power and time [12]. Permanganate has been widely studied as an oxidant with good stability and relatively low cost in water purification, but its stability hinders its reactivity in some cases [13]. Therefore, some researchers choose a composite process or additives for degradation to make up for shortcomings or assist operation of single process.

The development of emerging materials has also promoted AOPs for organic pollutants. Gholami et al. [14] investigated ultrasonic degradation catalyzed by a Fe-Cu-layered double hydroxide/biochar nanocomposite for cefazolin sodium, which had a 90% higher degradation efficiency than only ultrasonic treatment. In addition, the combination of two advanced oxidation technologies could have a synergistic effect, reducing the total cost while improving the total treatment effect. Periodate and persulfate were mixed with ultraviolet radiation and titanium dioxide nanoparticles to degrade and mineralize pirimicarb insecticide in aqueous media, with mineralization rates of 35% and 46%, respectively, after 60 min [15]. As a result, many AOPs have proven efficient in removing organic contaminants and have been vigorously developed. The purpose of this Special Issue is to collect cutting-edge results from recent research, investigate the degradation of organic pollutants by advanced oxidation technologies, and explore the mechanisms and impacts on the associated environment.

Peroxonosulfate (PMS) has the advantages of high redox potential, long half-life, and wide application range, attracting much attention in the field of pollutant treatment. Shi et al. (contribution 1) confirmed the synergistic effect of PMS and ferrate(VI) on the co-degradation of butylated hydroxyanisole (BHA). When T = 25 °C, the initial pH = 8.0, [BHA]_0_ = 100 μM, and [PMS]_0_:[Fe(VI)]_0_:[BHA]_0_ = 100:1:1, the degradation efficiency could reach 92.4% within 30 min, a 29.3% improvement over the sum degradation of the PMS system and Fe(VI) system. The main degradation pathways included hydroxylation, ring opening and coupling reactions, in which hydroxylation products were generated from •OH attacking BHA. Wang et al. (contribution 2) investigated the degradation performance of ciprofloxacin (CIP) in a magnetic graphene-oxide (MGO)-activated PMS system. The results showed that PMS could effectively remove CIP from an aquatic environment through the catalysis of stable MGO. •OH and •SO_4_^−^ generated in the MGO/PMS system were the main reaction species for oxidation, resulting in the effective destruction of CIP molecules. The Z-type heterojunction MnO_2_@g-C_3_N_4_ was used by Lu et al. (contribution 3) to catalyze the oxidation of tetracycline (TC) by PMS, and the degradation efficiency increased by about 38.7% compared with the g-C_3_N_4_/PMS system. Various characterization methods showed that the addition of MnO_2_ increased the absorption of g-C_3_N_4_ to visible light. Additionally, capture experiments showed that •OH and •SO_4_^−^ were still the main active species for TC degradation in the composite system.

As mentioned, sufficient studies have shown that free radicals play an important role in AOPs. Therefore, many researchers are trying to use and even produce free radicals to attack organic compounds and achieve degradation. Kang et al. (contribution 4) explored direct and indirect photolysis with free radicals (•OH, •NO_3_, and •SO_4_^−^) as active substances of Florfenicol (FLO) in an aqueous environment. The direct photolysis of FLO involved the cleavage of C-C/C-N/C-S bonds, while indirect photolysis mainly included OH addition, NO_3_ addition, and SO_4_ addition on the benzene ring. Indirect photolysis of FLO was more likely to occur than direct photolysis in the natural environment. Theoretical calculations were utilized by Sun et al. (contribution 5) to study the degradation mechanism and kinetics of Metronidazole (MNZ) in the presence of •OH and •SO_4_^−^. They found that the degradation pathways were mainly O_2_ addition, hydrogen abstraction, and bond breaking, and the most feasible reaction mechanism was that •OH and •SO_4_^−^ were added to the carbon atom attached to the NO_2_ group in the MNZ molecule. The rate constants of MNZ with •OH and •SO_4_^−^ were calculated as (3.54 ± 0.42) × 10^9^ and (2.74 ± 0.13) × 10^9^ M^−1^ s^−1^, respectively.

Plasma is also a feasible technology for effectively removing contaminants. •OH, •O, O_3_, H_2_O_2_, and other active species are generated by high-voltage discharge between two electrodes to decompose target molecules. Yao et al. (contribution 6) confirmed that the addition of cobalt oxyhydroxide (CoOOH) significantly improved the Methylene Blue (MB) degradation performance compared with a double dielectric barrier discharge system alone. In addition, with increased CoOOH dosage and discharge voltage, the removal rate and energy efficiency improved. In the catalytic system, the active species H_2_O_2_ and O_3_ were converted to the more oxidizing •OH, thus making the decomposition of the parent compound and intermediates more efficient. Li et al. (contribution 7) demonstrated that nanomaterial ZnO-Fe_3_O_4_ can effectively degrade ciprofloxacin (CIP) in chemical wastewater when combined with non-thermal plasma (NTP). The [ZnO]:[Fe_3_O_4_] ratio was determined as 14%:86%. The experimental results showed that •OH, •O_2_^−^, and ^1^O_2_ had certain effects on degradation. The main degradation pathways revealed by liquid chromatography–mass spectrometry were hydroxyl addition, hydroxyl substitution, and piperazine ring destruction.

With so many AOPs being used for contaminant removal, different methods and contaminants should be compared for optimal degradation and reduced toxicity. Sun et al. (contribution 8) employed a variety of theoretical calculation methods to analyze the degradation mechanisms and toxicity changes of fluoroquinolones (FQs) in different AOPs. Through the application of density functional theory and comparative molecular similarity index analysis, the relationship between the bond strength and molecular structure of FQs and their degradation products was elucidated, uncovering potential differences in bond dissociation energy. The prediction of toxicity offered a unique perspective for strategically utilizing, handling, and degrading FQs to prevent the formation of highly toxic compounds.

The ultimate goal of AOP-related research is to efficiently and reasonably solve the pollution problem in the real environment. Li et al. (contribution 9) explored the migration and transformation process of polycyclic aromatic hydrocarbons (anthracene (Ant), 9-chloroanthracene (9-ClAnt), benzopyrene (BaP), and chrysene (Chr)) in soil and their interactions with native communities. By the 56th day, BaP, Chr, and Ant contents were greatly reduced, but 9-ClAnt degradation was inhibited. During the degradation process, 19 intermediate compounds were identified, including hydroxylated and carboxylated compounds. During this period, the species diversity and relative abundance of soil bacterial and fungal communities also changed, including *lysosomes*, *Bacillus*, *pseudomonas*, and *Massilia* bacteria. Amacosta et al. (contribution 10) studied the treatment of paper industry wastewater by ozone oxidation and biodegradation. In this process, ozone oxidation was used as a pretreatment to convert parent compounds into low-molecular-weight ones. Subsequently, biodegradation decomposed the initial organic mixture, achieving a higher degree of mineralization.

The ten articles in this Special Issue explain the development and exploration of AOPs from different aspects, and contribute to our understanding of AOPs and emerging organic contaminant removal. These findings promote the efficient, green, and low-cost removal of environmental pollution to ensure the healthy life of human beings and the stable operation of ecosystems.
